# Endothelial Colony Forming Cells (ECFCs) in murine AKI – implications for future cell-based therapies

**DOI:** 10.1186/s12882-017-0471-3

**Published:** 2017-02-06

**Authors:** D. Patschan, K. Schwarze, B. Tampe, M. Zeisberg, S. Patschan, G. A. Müller

**Affiliations:** Clinic of Nephrology and Rheumatology, University Medicine Göttingen, Robert-Koch-Straße 40, 37075 Göttingen, Germany

## Abstract

**Background:**

In recent years, early Endothelial Progenitor Cells (eEPCs) have been proven as effective tool in murine ischemic AKI and in diabetic nephropathy. The mechanisms of eEPC-mediated vasoprotection have been elucidated in detail. Besides producing a diverse range of humoral factors, the cells also act by secreting vasomodulatory microvesicles. Only few data in contrast have been published about the role of so-called Endothelial Colony Forming Cells (ECFCs - late EPCs) in ischemic AKI. We thus aimed to investigate ECFC effects on postischemic kidney function over several weeks. Our special interest focused on endothelial-to-mesenchymal transition (EndoMT), peritubular capillary density (PTCD), endothelial alpha-Tubulin (aT - cytoskeletal integrity), and endothelial p62 (marker of autophagocytic flux).

**Methods:**

Eight to twelve weeks old male *C57Bl/6 N* mice were subjected to bilateral renal pedicle clamping for 35 or 45 min, respectively. Donor-derived syngeneic ECFCs (0.5 × 10^6^) were i.v. injected at the end of ischemia. Animals were analyzed 1, 4 and 6 weeks later.

**Results:**

Cell therapy improved kidney function exclusively at week 1 (35 and 45 min). Ischemia-induced fibrosis was diminished in all experimental groups by ECFCs, while PTCD loss remained unaffected. Significant EndoMT was detected in only two of 6 groups (35 min, week 4 and 45 min, week 6), ECFCs reduced EndoMT only in the latter. Endothelial aT declined under almost all experimental conditions and these effects were further aggravated by ECFCs. p62 was elevated in endothelial cells, more so after 45 than after 35 min of ischemia. Cell therapy did not modulate p62 abundances at any time point.

**Conclusion:**

A single dose of ECFCs administered shortly post-ischemia is capable to reduce interstitial fibrosis in the mid- to long-term whereas excretory dysfunction is improved only in a transient manner. There are certain differences in renal outcome parameters between eEPCs and ECFC. The latter do not prevent animals from peritubular capillary loss and they also do not further elevate endothelial p62. We conclude that differences between eEPCs and ECFCs result from certain mechanisms by which the cells act around and within vessels. Overall, ECFC treatment was not as efficient as eEPC therapy in preventing mice from ischemia-induced mid- to long-term damage.

## Background

Endothelial Progenitor Cells (EPCs) are heterogeneous in terms of origin and biological properties. A vast amount of EPC-related literature has been accumulated since their first description in 1997 [1]. Very early concepts described the cells as substitutes of damaged mature endothelial cells, suggesting a direct mechanism of vascular repair [1–3]. However, our understanding of EPC biology has fundamentally been changed over the last 10 years. It has become evident that the cells are represented by at least two major subpopulations, *early* and *late* Endothelial Progenitor Cells (eEPCs/lEPCs). The fundamental difference between the two lies in the fact that eEPCs display hematopoietic characteristics while lEPCs exclusively express endothelial but no hematopoietic marker molecules [4]. Meanwhile lEPCs have been defined as ‘true’ progenitors of endothelial cells, eEPCs in contrast should be recognized as ‘proangiogenic hematopoietic cells’ or simply as ‘proangiogenic cells’ (PACs) [5, 6].

Late EPCs may also be defined as Endothelial Colony-Forming Cells (ECFCs) [4, 5, 7–12]. In contrast to eEPCs/PACs, ECFCs mediate vascular repair in a more direct manner, by incorporating into the endothelial layer of damaged blood vessels. Nevertheless, Burger and colleagues identified another mechanism of ECFC action. Comparable to eEPCs, the cells secrete certain types of exosomes which may prevent rats from AKI if administered in a selective manner [7].

Acute kidney injury (AKI) remains a fundamental problem in the field of intensive care medicine in Europe and the US. Incidences and mortality rates have only mildly been improved during the last 20 years [13]. AKI patients suffer from significant short-term consequences that evolve during the first days after onset of acute kidney damage. Impaired excretion of water, solutes, and endogenous toxins cause serious alterations of cardiovascular and cerebral functions, respectively. The poor prognosis of AKI also ensues from the underlying disease or etiology. Thus, mortality may range from 30-50%, even if dialysis treatment has been initiated [14]. Another problem that arises in the mid- to long-term is an increased risk for chronic kidney disease (CKD). AKI is regularly associated with a loss of peritubular capillaries and the accumulation of connective tissue in the interstitium [15–19]. As a matter of fact, interstitial fibrosis better correlates with the risk of CKD progression than glomerular sclerosis. The mechanisms perpetuating kidney fibrosis are complex and different cell types have been shown to undergo a process of mesenchymal transdifferentiation in CKD, namely tubular epithelial cells (Epithelial-to-Mesenchymal Transition – EMT [20]). Another source of mesenchymal matrix proteins are mature endothelial cells within peritubular vessels (capillaries, arterioles). Investigations performed by the groups of Goligorsky and Kalluri [21, 22] revealed Endothelial-to-Mesenchymal Transition (EndoMT) as relevant cause of interstitial fibrosis in different disease models of CKD. Investigations performed by our group confirmed these findings [23, 24]. As a matter of fact, the prognosis of AKI has not substantially been improved since the early 1990s, although a lot of progress has been achieved in the fields of dialysis treatment and intensive care medicine. Therefore new therapeutic strategies are urgently needed. In recent years, stem/progenitor cell based therapies have been studied more in detail and promising results were acquired by using mesenchymal stem cells (MSCs [25, 26]). Another interesting cell population is represented by so-called induced pluripotent stem cells (iPS) [27–29]. Whether these or other cell-types will truly be established as future therapeutic measures in AKI or not remains debatable. Nevertheless, even if any transfer of cell-based strategies should fail, scientists and clinicians can potentially acquire new knowledge about AKI and possible therapeutic targets by studying the mechanim of (stem/progenitor) cell-mediated AKI protection.

EPCs, and in particular early EPCs have been used for anti-ischemic treatment in numerous experimental disease models, such as ischemic heart, cerebrovascular, and peripheral artery disease, respectively [30–35]. In addition, several studies showed the cells to be effective in experimental AKI as well [36–38]. Own investigations focused on strategies for improving efficacy of eEPC-treatment in murine AKI. We were able to identify several substances that are capable to augment renoprotective cell effects [23, 39–42]. Lately, we analyzed mid-term consequences of AKI treatment with a single dose of native eEPCs, systemically applied at the time of reperfusion. The study showed robust improvement of serum creatinine levels, interstitial fibrosis, EndoMT and peritubular capillary density [43]. Thus, EPCs have been established as reliable tool in AKI in the short- and mid-term. It needs nevertheless to be mentioned that endogenous early EPCs and ECFCs are most likely not relevant for direct endothelial repair in AKI. Sradnick and colleagues lately published a manuscript on the topic which substantially questioned the role for extrarenal progenitor cells in endothelial regeneration [44]. This study however does not truly conflict with our past and the current investigation(s) that primarily intend to establish EPCs as therapeutic tool in AKI.

The data on ECFCs for AKI therapy are quite limited. The only study performed so far was published by Burger and colleagues [7]. We therefore aimed to investigate ECFCs effects in murine ischemic AKI. Our particular interest focused on parameters of interstitial kidney damage, such as fibrosis, EndoMT, endothelial autophagy, and endothelial alpha-tubulin, a marker of cytoskeletal integrity.

## Methods

### Animals models

As in previous investigations, the present animal study protocol was in accordance with the guidelines of the German Institute of Health Guide for the Care and Use of Laboratory Animals and approved by the Institutional Animal Care and Use Committee. All experiments were performed in male, 8–12 weeks old C57BL/6 N mice, which were originally obtained from Jackson Labs (Bar Harbor, ME, USA) and bred in the local animal facility of the Göttingen University Hospital. All animals were separately caged with a 12:12-h light–dark cycle and had free access to water and chow throughout the study.

### Surgical procedures

The surgical procedure has extensively been performed and published several times before [23, 43, 45]. We decided to apply two different ischemic conditions (35 and 45 min) in order to mimic AKI of different severity. Animals were sacrificed (puncture of the heart and diaphragma cut) and analyzed 1, 4, and 6 weeks later, respectively.

### Culture of mouse-derived early and late endothelial progenitor cells

Early EPCs were cultured according to an established protocol [43]. In detail, mononuclear cells (MNCs) were enriched by density gradient centrifugation using Biocoll solution (Biochrom, Berlin, Germany) from peripheral blood and spleen cell extracts. MNCs were pooled from blood and spleen in order to increase the total number of cells available for therapy. Immediately following isolation, mononuclear cells were mixed and 4 × 10^6^ cells were plated on 24-well culture dishes coated with human fibronectin (Sigma, St Louis, MO) and maintained in endothelial cell medium-2 (EGM-2 - Clonetics, Lonza, Walkersville, MD, USA) supplemented with endothelial growth medium (EGM) Single-Quots containing 5% FCS. For VE-Cadherin, CD14, and CD45 staining, cells were grown on fibronectin-coated glass bottom slides, the detailed procedure will be described below. After 4–5 days of culture, eEPCs were identified by the uptake of DiI-labeled acetylated low-density lipoprotein (acLDL) (Invitrogen, Carlsbad, CA, USA) and binding of FITC-labeled BS-1 lectin (BS-1) (Sigma Diagnostics, St. Louis, MO).

Late EPCs were cultured according to the following procedure: peripheral mononuclear cells (4 × 10^6^), isolated by density centrifugation (see above) were cultured on fibronectin-coated glass bottom slides in EGM-2 for an average of 9 weeks. After washing the slides with PBS, cells were fixated using 3.7% PFA in PBS for 15 min at room temperature. After cell permeabilization with 0.1% Triton X100 (20 min at room temperature) 1% milk powder was applied for 30 min at room temperature. Primary incubation was performed overnight at 4 °C using the following antibodies: anti-mouse VE Cadherin, host rabbit (Abcam, ab33168, 1:200), anti-mouse CD45, host rat (Abcam, ab23910, 1:50), anti-mouse CD14, host mouse (Abcam, ab182032, 1:100). After cell washing using PBS, secondary incubation was performed for 1 h at room temperature with the following antibodies: anti-rabbit 488 (1:400), anti-rat 594 (1:400), anti-mouse 594 (1:400) (all secondary antibodies were purchased from Jackson Dianova). Finally, the nuclei were stained with DAPI solution.

Stained cells were analyzed using an inverted fluorescence microscope IX-71 (Olympus Deutschland GmbH, Hamburg, Germany) equipped with the appropriate excitation and emission filters (AHF Analysentechnik, Tübingen, Germany). Images of respective fluorescence channels were recorded as single high resolution 16bit b/w images using a F-View II ext. Camera (Olympus Deutschland GmbH, Hamburg, Germany). The images from every fluorescence channel were then automatically merged using the MFIP-module of the CELL-F® software.

### Morphological analyses

The methodical procedures for quantification of fibrosis, PTCD, EndoMT, and aT have been described in detail recently [43]. Kidney fibrosis was examined in formalin fixated, paraffin-embedded tissue sections after Masson Trichrome staining. The amount of collagen deposition (blueish area) was semiquantitatively assessed by assigning grade 1 (mild), 2 (moderate) or 3 (severe). EndoMT and endothelial aT were also evaluated in formalin fixated, paraffin-embedded tissue sections after deparaffinization, followed by incubation in 3% H_2_O_2_ for 10 min. After citrate-buffer treatment (microwave, 5 times 3 min, pH 6.0) sections were stained with rat anti-mouse CD31 (PECAM-1 - CloneSZ31, Dianova), and with either mouse anti-acetylated alpha Tubulin (abcam - ab24610) or rabbit anti-SMA (EMELCA) for primary incubation and with Alexa Fluor 488 goat anti-rabbit IgG (Dianova), Alexa Fluor 594 goat anti-rat IgG (Dianova), and Alexa Fluor 488 anti-mouse IgG (Dianova) for secondary incubation, respectively. Primary incubation was performed overnight at 4 °C while secondary incubation was performed for 1 h at room temperature. p62 staining was performed as described recently [45]. Rabbit anti p62 (abcam ab91526) was used for primary incubation (4 °C, overnight), followed by secondary incubation with anti rabbit 488 (Jackson ImmunoResearch) for one hour at RT. To visualize the nuclei, tissue sections were counterstained with DAPI. Three view fields per kidney were analyzed for co-localization of either SMA, or aT, or p62 and CD31 using ImageJ software. Confocal analyses were performed with the Zeiss® LSM780 microscope and with the Olympus® FLUOVIEW FV1000 microscope.

### Renal function

Serum creatinine concentration was measured using a commercially available kit (Creatinin, Jaffé, Labor und Technik, Eberhard Lehmann, LT-CR0121, Berlin, Germany) according to the manufacturer’s protocol.

### Statistical analysis

The results were expressed as mean ± SEM. The means of two populations were compared by Student’s *t* test. Differences were considered significant at *p* < 0.05.

## Results

In all analyzed categories, comparisons were made to untreated control and to post-ischemic experimental groups that have previously been used in another investigation related to early EPCs. The study has lately been published separately in the *American Journal of Physiology – Renal Physiology* [43].

Ten animals were included into each group, no animals died during follow-up.

### Phenotypical characteristics of syngeneic murine eEPCs and lEPCs

**Fig. 1:**
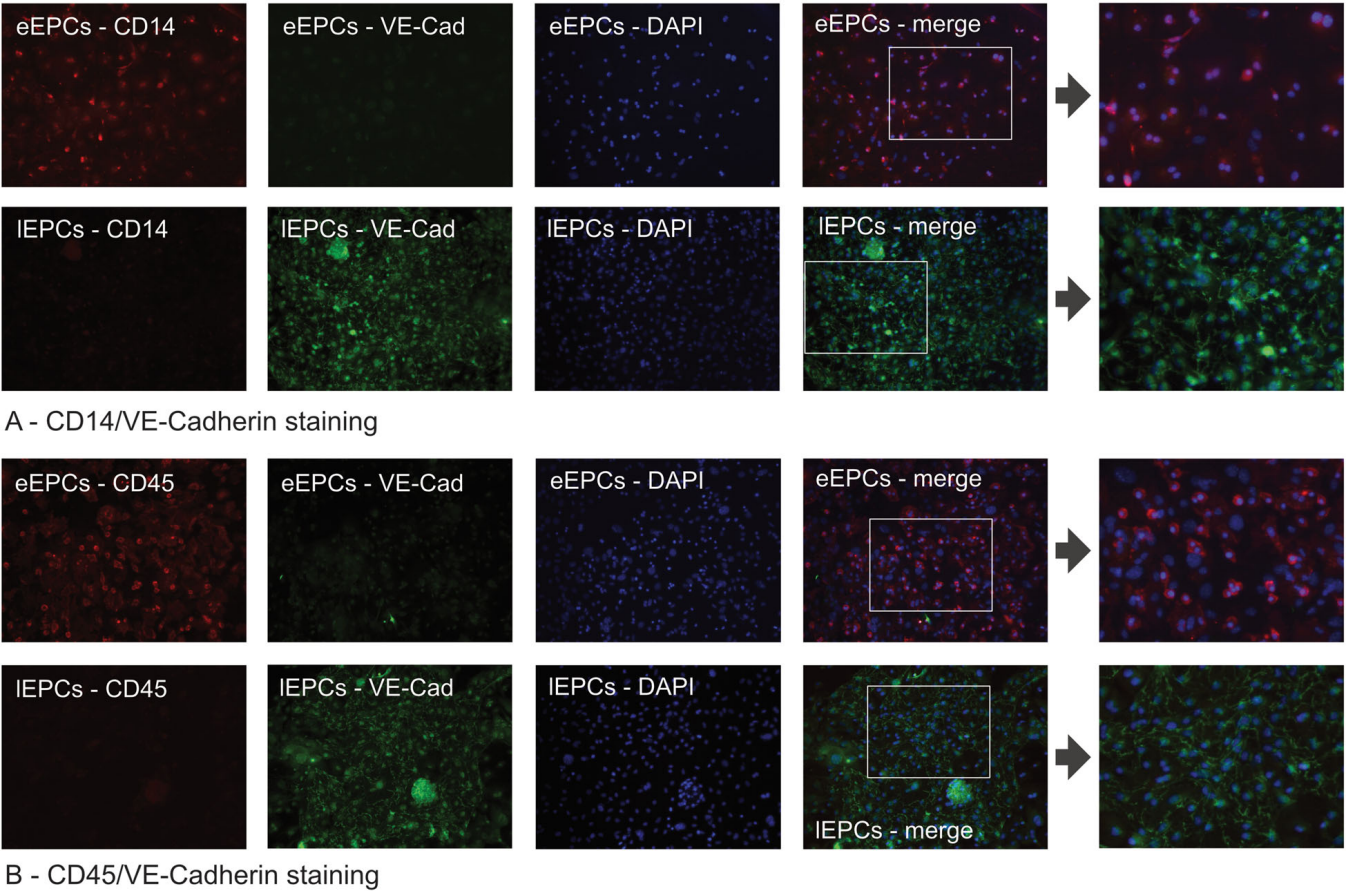
Surface marker expression patterns of cultured late (lEPCs) as opposed to early Endothelial Progenitor Cells (eEPCs). Panel A depicts combined staining of CD14 and VE-Cadherin, B shows staining of both cell types for CD45 and VE-Cadherin. Early EPCs expressed both hematopoietic markers (CD14 and CD45) whereas VE-Cadherin was not detectable at all. Late EPCs in contrast were strongly VE-Cadhein positive but negative for the other proteins, respectively (magnifications × 20 except the images behind the arrows: ×80; red in panel **a**: CD14, in panel **b**: CD45; green in both panels: VE-Cadherin; blue in both panels: DAPI – nuclei; the white rectangles in panel **b** show magnified areas behind the arrows)

Both, early and late EPCs were cultured according to established protocols [7, 43]. In order to differentiate between the two populations we defined early EPCs as VE-Cadherin^−^/CD14^+^ or CD45^+^ cells and late EPCs as VE-Cadherin^+^/CD14^−^ or CD45^−^ cells. This decision was made in adaption to the observations by Burger and colleagues who detected neither CD14 nor CD45 on late EPCs (ECFCs – Endothelial Colony Forming Cells [7]). Figure 1 shows the results of fluorescence analysis. Early EPCs expressed both hematopoietic markers (CD14 and CD45), whereas VE-Cadherin was detected with very low intensities. Late EPCs in contrast were strongly VE-Cadhein positive but negative for the CD14 and CD45, respectively.

### Serum creatinine

**Fig. 2:**
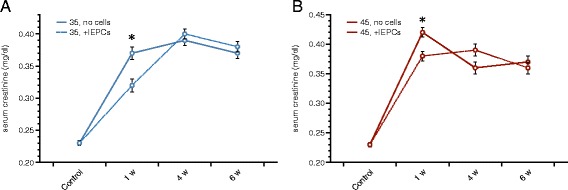
Postischemic serum creatinine in all experimental groups (**a**: 35 min, **b**: 45 min). Renal ischemia significantly increased creatinine levels after 35 and 45 min of ischemia. Concentrations remained elevated throughout the whole postischemic period (until week 6). Systemic administration of native lEPCs improved excretory kidney function exclusively at week 1 (35 and 45 min of ischemia) (Data as mean ± SEM, ✻: *p* < 0.05)

The baseline serum creatinine in untreated controls was 0.23 ± 0.004 mg/dl. Postischemic creatinine levels significantly increased under both experimental conditions (35 and 45 min of ischemia). They remained elevated throughout the whole period of post-ischemic analysis (1, 4, and 6 weeks). Serum creatinine concentrations in mg/dl: 35 min - 1 week(s) (w) 0.37 ± 0.01; 4 w 0.39 ± 0.008; 6 w 0.37 ± 0.008; 45 min - 1 w 0.42 ± 0.008; 4 w 0.36 ± 0.01; 6 w 0.38 ± 0.01. All *p*-values as compared to controls were below 0.0001. Administration of a single dose of 0.5 × 10^6^ native syngeneic lEPCs at the end of ischemia significantly improved serum creatinine levels at week 1. This effect occurred after both, 35 and 45 min: 35 min 1 w + lEPCs 0.32 ± 0.01 (*p*-value vs. ischemia without cells 0.007); 45 min 1 w + lEPCs 0.38 ± 0.008 (*p*-value vs. ischemia without cells 0.01). Cell therapy did not improve creatinine levels at later time points (4 and 6 weeks - numerical data not given). Figure 2 summarizes the post-ischemic serum creatinine concentrations in all experimental groups.

### Interstitial fibrosis and EndoMT

Next, we aimed to analyze interstitial fibrosis and mesenchymal transdifferentiation of intrarenal endothelial cells (EndoMT), the latter being reflected by colocalization of CD31 and aSMA in small peritubular arterioles [23, 24]. Fibrosis was assessed in a semiquantitative manner (arbitrary units).

**Fig. 3:**
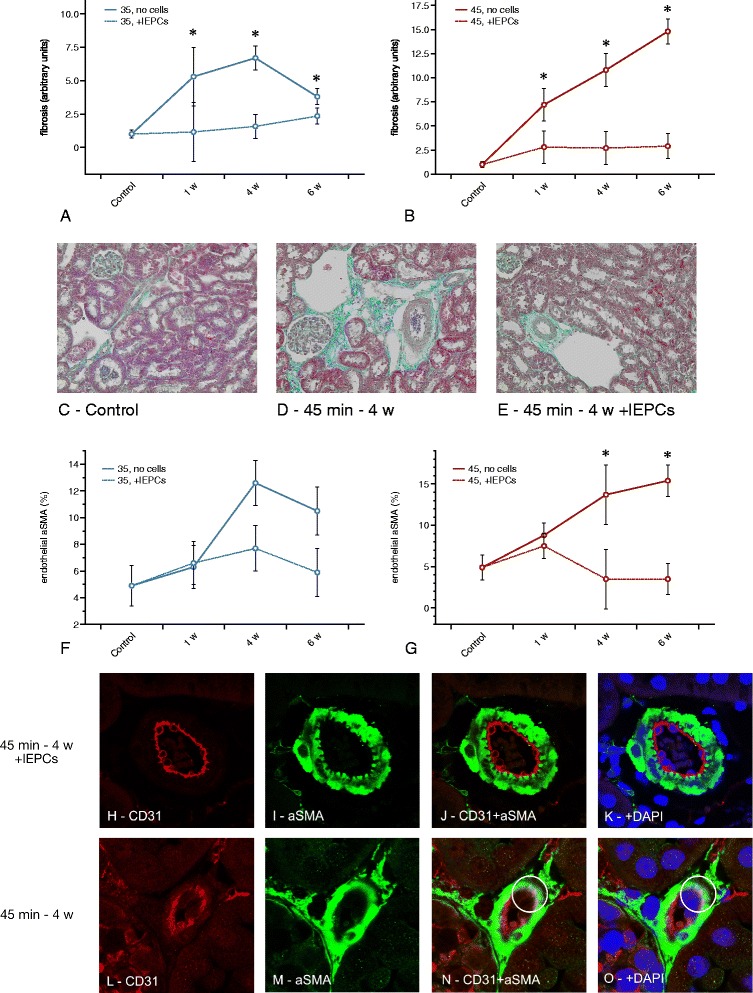
Renal fibrosis and EndoMT in the respective groups at 1, 4, and 6 weeks post-ischemia. As pointed out in the text, significant matrix deposition occurred under all experimental conditions. In addition, cell administration reduced fibrosis after 35 and 45 min at weeks 1, 4, and 6, respectively (**a** – **e**). **f** and **g** show the results of mesenchymal analysis. Significant EndoMT exclusively occurred in the 35 min group at week 4 and in the 45 min group at week 6. Cell treatment reduced endothelial aSMA expression at week 4 (45 min) and at week 6 (45 min). Images (**h** - **o**) display detailed aSMA staining patterns within the endothelium. **h** – **k** show endothelial and mesenchymal staining at 45 min with cell therapy (4 weeks), (**l** – **o**) depict the same staining without cell administration. Images (**n**) and (**o**) allow to identify areas of CD31/aSMA co-expression in a detailed manner (white circles). In contrast, lEPC therapy stabilized the endothelial-muscular borderzone distinctively. The latter was less accentuated in animals which did not receive any cells at all (magnifications × 40 in **c** – **e**; ×160 in **h** - **o**; green in all microscopic images: aSMA; red: CD31; blue: nuclei; Data as mean ± SEM, ✻: *p* < 0.05)

Fibrosis: The baseline value in untreated controls was 1.0 ± 0.3. As a matter of fact, significant fibrosis occurred under all experimental conditions at every time point: 35 min - 1 week(s) (w) 5.3 ± 2.3; 4 w 6.5 ± 0.9; 6 w 3.8 ± 0.6; 45 min - 1 w 7.1 ± 1.7; 4 w 10.8 ± 1.7; 6 w 14.8 ± 1.3. The *p*-values as compared to controls were: 35 min – 1 w 0.04; 35 min – 4 w <0.0001; 35 min – 6 w 0.0004; 45 min – 1 w 0.002; 45 min – 4 w <0.0001; 45 min – 6 w <0.0001. Cell therapy diminished matrix deposition under both experimental conditions at every time point: 35 min - 1 w + lEPCs 1.1 ± 0.1; 4 w + lEPCs 1.5 ± 0.1; 6 w + lEPCs 2.3 ± 0.2. The *p*-values for every time point were (with vs. without cell administration): 1 w 0.04; 4 w <0.0001; 6 w 0.03; 45 min - 1 w + lEPCs 2.8 ± 0.3; 4 w + lEPCs 2.7 ± 0.3; 6 w 2.8 ± 0.5; the *p*-values were: 1 w 0.01; 4 w <0.0001; 6 w <0.0001 (Fig. 3).

Although ischemia increased matrix deposition, EndoMT (aSMA in CD31+ cells in %) was detected in a significant manner only at week 4 in the 35 min group (12.6 ± 1.7% vs. controls 4.9 ± 1.5%; *p* < 0.0001) and at week 6 in the 45 min group (15.3 ± 1.9% vs. controls 4.9 ± 1.5%; *p* < 0.0001). Cell treatment diminished EndoMT at 4 and 6 weeks but exclusively in the 45 min group: 4 w + lEPCs 3.5 ± 0.9% and 6 w + lEPCs 3.5 ± 0.7%; the *p*-values vs. ischemia without cell therapy were 4 w 0.01 and 6 w 0.0001. Figure 3 summarizes the results of mesenchymal analysis.

### Peritubular capillary density and endothelial alpha-Tubulin

**Fig. 4:**
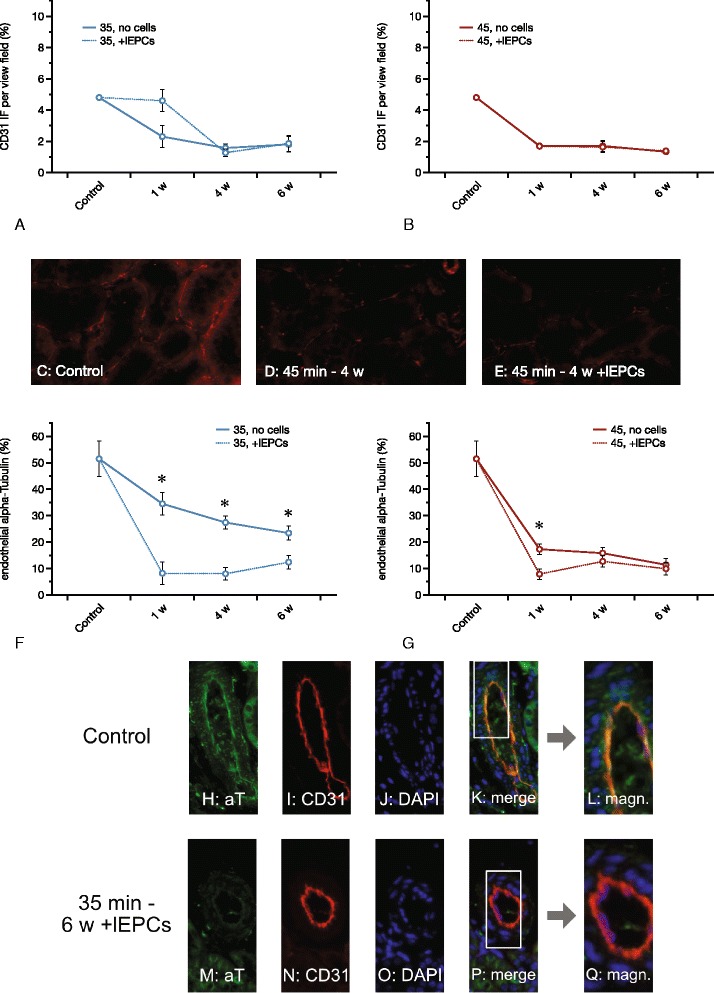
Peritubular capillary density (PTCR) and endothelial alpha-Tubulin (aT) expression. PTCR was evaluated by measuring CD31 immunofluorescence per cortical area. **a** and **b** show PTCR dynamics under the different experimental conditions with (**a**) for the 35 and B for the 45 min groups. In comparison to the control group, ischemia significantly reduced PTCR in a prolonged manner (week 1 to 6). The only exception was the 35 min group at week 1. Contrasting to earlier results published recently [43] late EPC therapy did not improve capillary densities in any of the groups. **c** – **e** show representative images of CD31 analysis in controls as compared to two experimental groups. **f** and **g** show endothelial aT levels with versus without lEPC therapy with (**f**) for the 35 and (**g**) for the 45 min groups. As mentioned in the text, endothelial aT decreased under all experimental conditions with one exception: 35 min, week 1. Cell therapy further aggravated aT reduction with two exceptions: 45 min, weeks 4 and 6. **h** – **q** give detailed impressions of endothelial aT dynamics. **h** – **l** show single stained sections for aT (green – **h**), CD31 (red – **i**), nuclei (blue – **j**), and RGB merge (all three colors – **k**) in the control group. **l** magnifies the white area in (**k**) fourfold. **m** – **q** display respective single-stained sections from the 35 min 6 weeks + lEPC group (magnifications × 40 in **c** – **e** and in **h** – **k**/**m** - **p**; ×160 in **l** and **q**; yellow in **k**, **l**, **p**, and **q**: CD31/aT overlap; Data as mean ± SEM, ✻: *p* < 0.05)

Peritubular capillary rarefaction (PTCR) has been shown to occur after ischemia and to promote progression towards CKD [17, 18, 46]. PTCR was assessed by quantification of peritubular CD31 immunofluorescence per area (in %). Controls displayed a mean percentage of 4.8 ± 0.01. PTCR declined under all experimental conditions with one exception (35 min - 1 w): 35 min - 1 w 2.4 ± 0.8; 4 w 1.5 ± 0.2; 6 w 1.8 ± 0.5; 45 min - 1 w 1.7 ± 0.1; 4 w 1.7 ± 0.3; 6 w 1.3 ± 0.1. The *p*-values vs. controls were: 35 min - 1 w 0.09; 4 w 0.006; 6 w 0.02; 45 min - 1 w 0.001; 4 w 0.01; 6 w 0.001. Cell therapy did not modulate PTCR at any time point, neither after 35 nor after 45 min of ischemia (Fig. 4).

In a recent study, endothelial acetylated alpha-Tubulin (aT) has been proposed to serve as one possible marker of endothelial cilia integrity [43], although it is not specific for cilia but can also be detected in various other microtubule structures. Flow-treatment of cultured mature endothelial cells reduced percentages of cilia^+^ cells accompanied by lower cellular abundances of aT. Cellular expression of alpha-Smooth Muscle Actin (aSMA), a marker of mesenchymal transdifferentiation increased [43]. These effects were completely reversible after incubation of the cells with supernatant from untreated murine eEPCs. Analyses of postischemic kidneys showed reduced EndoMT to parallel with lower endothelial expression of aT as well. These effects were partially augmented by cell treatment with eEPCs [43]. Thus, lower endothelial aT was discussed to reflect an ‘antifibrotic’ state. In the current study, dynamics of endothelial aT were comparable to those mentioned in the other study: endothelial aT declined under almost all experimental conditions (results given as percentages of the endothelial surface area, stained positive for aT; *p*-values indicate differences as compared to controls; controls 51.5 ± 6.7%; 35 min - 1 w 34.4 ± 4.3%, *p* = 0.06; 35 min - 4 w 27.4 ± 2.4%, *p* = 0.007; 35 min - 6 w 23.5 ± 2.6%, *p* = 0.003; 45 min - 1 w 17.3 ± 2.1%, *p* = 0.0006; 45 min - 4 w 15.8 ± 2.2%, *p* = 0.0005; 45 min - 6 w 11.3 ± 2.6%, *p* = 0.0006), these effects were further aggravated by lEPC treatment in all 35 min groups (*p*-values indicate differences as compared to animals without cell therapy; 35 min - 1 w + lEPCs 8.2 ± 2.6%, *p* = 0.0004; 35 min - 4 w + lEPCs 8 ± 1.1%, *p* < 0.0001; 35 min - 6 w + lEPCs 12.4 ± 2%, *p* = 0.009) and in the 45 min group at week 1 (45 min - 1 w + lEPCs 7.9 ± 1.8%, *p* = 0.007) (Fig. 4).

### Endothelial p62

**Fig. 5:**
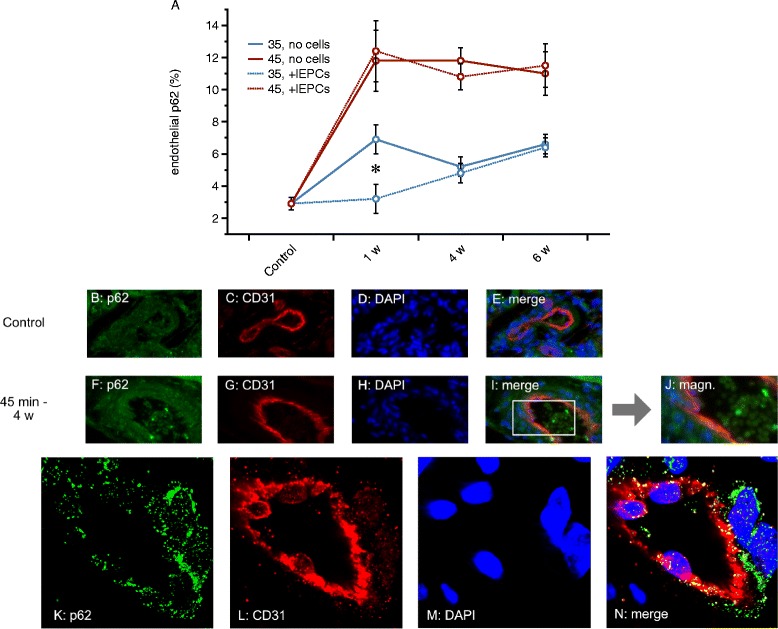
Endothelial p62 expression. Firstly, ischemia induced endothelial p62 in all experimental groups. Nevertheless, levels were always higher in the 45 than in the 35 min groups. Secondly, cell therapy failed to modulate endothelial p62 at any given timepoint with the only exception of 35 min at week 1 (**a**). **b** – **n** display representative images from controls and several experimental groups. The white rectangle in (**i**) surrounds areas of CD31/p62 colocalization. Images (**k** – **n**) show the results from confocal co-localization analysis with the respective monochromatic and one merged image(s). **k** and **n**: p62 shows a punctuated staining pattern within CD31+ cells but also within adjacent vascular wall cells (magnifications × 40 in **b** - **i**; ×160 in **j**; ×630 in **k** - **n**; green in all microscopic images: p62; red: CD31; yellow: CD31/p62 overlap; blue: nuclei; Data as mean ± SEM, ✻: *p* < 0.05)

In recent years, autophagy (AP) has increasingly been recognized as endogenous mechanisms of cellular self-defense. Recently, defective autophagy has been proposed to aggravate EndoMT in cultured endothelial cells [47]. The methodical approaches for visualizing AP in cultured cells/tissues have extensively been reviewed in the past [48, 49]. AP involves several subcellular fusion events ultimately resulting in the generation of autophagosomes (APS). Several marker molecules for AP monitoring have been established in the past, among those are LC3-II and the protein p62. Increased AP is usually associated with increased cellular abundance of LC3-II and lower availability of p62 [50]. However, elevated p62 levels have also been linked to increased flux through the autophagocytic cascade [48, 50]. We therefore aimed to investigate endothelial p62 in all experimental groups. In comparison to untreated controls endothelial p62 expression was significantly increased in every experimental group post-ischemia (*p*-values show differences as compared to controls; controls 2.9 ± 0.4%; 35 min - 1 w 6.9 ± 0.8%, *p* = 0.0003; 35 min - 4 w 5.2 ± 0.6%, *p* = 0.006; 35 min - 6 w 6.5 ± 0.6%, *p* < 0.0001; 45 min - 1 w 11.8 ± 1.9%, *p* < 0.0001; 45 min - 4 w 11.8 ± 0.8%, *p* < 0.0001; 45 min - 6 w 11 ± 1.3%, *p* < 0.0001). Secondly, endothelial p62 was lower in the 35 than in the 45 min groups at every given time point (35 min – 1 w vs. 45 min - 1 w *p* = 0.01; 35 min – 4 w vs. 45 min - 4 w *p* < 0.0001; 35 min – 6 w vs. 45 min - 6 w *p* = 0.002). Administration of native lEPCs did not modulate endothelial p62 with one exception: 35 min - 1 w + lEPCs 3.2 ± 0.5%; 35 min - 4 w + lEPCs 4.8 ± 0.6%; 35 min - 6 w + lEPCs 6.4 ± 0.6%; 45 min - 1 w + lEPCs 12.3 ± 1.6%; 45 min - 4 w + lEPCs 10.8 ± 1.4%; 45 min - 6 w + lEPCs 11.4 ± 1.4%. The *p*-value between 35 min - 1 w and 35 min - 1 w + lEPCs was 0.008. Figure 5 summarizes the results.

## Discussion

The current study is one of two investigations performed so far that analyzed the therapeutic role of Endothelial Colony Forming Cells (ECFCs) in experimental AKI. We chose two different ischemic periods which were not closely related to each other (35 and 45 min). We intended to induce ischemic AKI of variable severity. Nevertheless, the difference of 10 min may also allow to speak about to different AKI entities (mild versus moderate/severe AKI). In 2015, Burger and colleagues [7] evaluated consequences of i.v. ECFC administration in murine ischemic AKI, showing short-term (24 and 72 h) renoprotective effects of the cells. Similar results were obtained by exclusively injecting ECFC-derived exosomes. Our study was designed in order to analyze AKI mid-term outcomes after injection of a single dose of ECFCs at the time of reperfusion. We found improved kidney function at week 1 after ischemia, diminished kidney fibrosis in all experimental groups, significantly reduced EndoMT in two of 6 groups (45 min, 4 and 6 weeks), no preservation of PTCD at all, and decreased endothelial aT levels in all of the 35 min but only in one of the 45 min groups. Finally, endothelial p62, a marker of autophagocytic flux was analyzed. Cell therapy did not modulate p62 at all. We lately published a manuscript about the effectiveness of early EPCs in the same context [43]. Effects on kidney function were comparable and kidney fibrosis was also diminished in (almost) all groups.

Nevertheless, some differences with regard to PTCD, EndoMT, and endothelial aT shall be discussed. Firstly, ECFCs as opposed to eEPCs were not capable to prevent peritubular capillary loss. Several articles by Basile and Goligorsky discussed PTCD loss as critical event in AKI-related chronic kidney dysfunction [15–18, 46]. Cantaluppi and colleagues showed that EPC-derived microvesicles alone may prevent the kidney from post-AKI capillary rarefication, thus potentially reducing the risk for chronic kidney disease (CKD) [36]. The study by Burger et al. in contrast did not investigate animals later than 72 h after the ischemic insult [7]. Before any conclusion may be drawn, regarding not only PTCD but also EndoMT and endothelial aT abundances it is important to discuss cellular mechanisms of EPC(eEPC and lEPC) action within the (peri)vascular microenvironment. The initial description of EPCs by Asahara suggested a direct mechanism of vascular repair [1]. According to this particular concept, cells incorporate into the intimal layer of blood vessels in order to substitute damaged mature endothelial cells. Meanwhile, our understanding of EPC-mediated vasoprotection has significantly been expanded. Early EPCs predominantely act by indirect mechanims including the release of multiple humoral factors and of vasomodulatory microvesicles [4, 36, 51]. Vascular cell incorporation occurs sporadically, this has been confirmed by own studies as well [39, 40]. In recent years, early EPCs have therefore been proposed as hetergogeneous population of myelomonocytic cells or simply as proangiogenic cells (PACs) [19]. ECFCs or late EPCs on the other hand are most likely *true* progenitors of the endothelium, characterized by significant vessel-formation capacity both in vitro and in vivo [4, 52]. The lately published study by Burger et al. [7] was the first to describe indirect ECFC-mediated effects. Comparable to early EPCs, ECFCs were shown to secrete certain types of small vesicles (exosomes) which are capable to act tissue-protective per se. Nevertheless, no study showed that the cells act modulatory by producing humoral factors as well. Such absence of a specific ´secretome´ may explain outcome differences between eEPCs and ECFCs. In theory one may argue that stabilization of the capillary network predominantely results from indirect (humoral) effects of EPCs and those are (to our knowledge) not mediated by ECFCs. The same conclusion may be applicable with regard to discrepancies in EndoMT and endothelial aT. While early EPCs diminished EndoMT at week 4 post-ischemia (35 min) and at week 6 (45 min), ECFCs reduced mesenchymal transition at week 4 and 6 (45 min respectively). ECFCs also reduced endothelial aT, even in more groups than in the other study related to eEPCs [43]. In our latest study [43] we proposed a dynamic cascade of eEPC-mediated cytoskeletal (acetylated alpha-Tubulin) reorganization with subsequent stabilization of the endothelial ciliome, ultimately resulting in diminished EndoMT. Although ECFCs reduced aT abundances in even more groups that eEPCs, the current findings partly support our previous hypothesis. Nevertheless, it appears premature to generally propose reduced endothelial levels of acetylated aT as endothelial-protective mechanism. An association between endothelial aT and EndoMT may exist, but a mechanistic relationship between aT and EndoMT/reduction of endothelial dysfunction in AKI cannot be claimed with certainty at the moment. Further studies must particularly address dynamics of endothelial aT and functional alterations such as endothelial permeability and viability.

Endothelial p62 increased after ischemia with even more intensive staining patterns after 45 min of ischemia. Similar findings have been reported in another study from 2015 [45]. The protein p62 has been suggested as marker of autophagocytic flux [48]. As opposed to eEPCs [45], ECFCs did not further increase endothelial p62. There was no difference between ischemia without and ischemia with cell therapy. In addition, there was no association between p62 dynamics and cell-mediated modulation of EndoMT. Whether in our study elevated endothelial p62 reflected a persistently increased flux through the autophagocytic cascade or not remains debatable at the moment. Regarding our findings, it is however impossible to conclude that both events, autophagy and EndoMT were significantly linked to each other since cell therapy inhibited EndoMT while p62 remained unaffected. We once again suppose that differences between eEPC- and ECFC-mediated effects on parameters of endothelial integrity most likely result from differences in cellular mechanisms of action.

Current own investigations extensively analyze the secretome of eEPCs and are also intended to identify paracrinice effects of ECFCs. We thus hope to further clarify some discrepancies between the two studies.

Our general conclusion is that ECFCs are capable to protect the postischemic kidney from persistent damage. We must nevertheless also conclude that the cells per se are less potent than eEPCs in this context. Particularly the missing effects on PTCD are critical in terms of CKD-progression. The differences between eEPCs and ECFCs are most likely attributed to the individual mechanisms by which these cells act around and within vessels. A deeper understanding of the processes involved will hopefully help to introduce EPCs or exclusively the cells’ modes of action (humoral factors, microvesicular structures) into the therapeutic AKI management. Finally, it has to be mentioned that in the current study cells were injected only once. The process of postischemic kidney repair may last for several days to weeks (or even months). Further studies are needed to evaluate the efficacy of alternative cell administration protocols (e.g. cell injection prior to and post-ischemia, repeated cell injection after ischemia). In addition, a transgenic model of constitutive endothelial cell tracing could serve as tool for assessing engraftment of early EPCs and ECFCs in the ‘therapeutic situation’.

## Conclusions

Our general conclusion is that a single dose of ECFCs administered shortly post-ischemia is capable to reduce interstitial fibrosis in the mid- to long-term whereas excretory dysfunction is improved only in a transient manner.

We must nevertheless also conclude that the cells per se are less potent than eEPCs in this context. Particularly the missing effects on PTCD are critical in terms of CKD-progression.

The differences between eEPCs and ECFCs are most likely attributable to the individual mechanims by which the cells act around and within vessels.

Regarding the previous conclusion we particularly conclude that eEPCs most likely exihibt stronger indirect effects, mediated by microvesicles/certain humoral factors. These may account for discrepancies between outcome parameters (EndoMT, aT, p62).

A deeper understanding of the processes involved will hopefully help to intro- duce EPCs or exclusively the cells’ modes of action (humoral factors, microvesicular structures) into the therapeutic AKI management.

## Abbreviations

acLDL: acetylated Low Density Lipoprotein
AKI: Acute kidney injury
aSMA: Alpha-smooth muscle actin
aT: Alpha-Tubulin
BS-1: Bandeiraea simplicifolia-1
CKD: Chronic kidney disease
DAPI: Diamidin-2-phenylindole
ECFCs: Endothelial colony forming cells
EBM-2: Endothelial growth basal medium-2
eEPCs: Early endothelial progenitor cells
EndoMT: Endothelial-to-mesenchymal Transition
lEPCs: Late Endothelial Progenitor Cells
MNCs: Mononuclear cells
PBS: Phosphate buffered saline
PFA: Paraformaldehyde
PTCD: Peritubular capillary density
